# Revealing the Mechanism of Protein Degradation in Postmortem Meat: The Role of Phosphorylation and Ubiquitination

**DOI:** 10.3390/foods14020184

**Published:** 2025-01-09

**Authors:** Xinran Zhao, Saisai Wu, Chi Ren, Yuqiang Bai, Chengli Hou, Xin Li, Zhenyu Wang, Dequan Zhang

**Affiliations:** Institute of Food Science and Technology, Chinese Academy of Agricultural Sciences/Key Laboratory of Agro-Products Quality & Safety in Harvest, Storage, Transportation, Management and Control, Ministry of Agriculture and Rural Affairs, Beijing 100193, China; xrzhao2021@163.com (X.Z.); wssaxiu1736@163.com (S.W.); candymce@163.com (C.R.); yuqiangbai_1844@163.com (Y.B.); houchengli@caas.cn (C.H.); caasjgsmeat2021_1@126.com (Z.W.); dequan_zhang0118@126.com (D.Z.)

**Keywords:** meat tenderness, protein posttranslational modifications, AMPK, E3 ubiquitin ligase

## Abstract

The aim of this study was to investigate the possible effects of phosphorylation and ubiquitination on the degradation of myofibrillar proteins in mutton with different tenderness. The *longissimus thoracis lumborum* muscles were chosen and divided into tender and tough groups (*n* = 9), and then stored at 4 °C for 1 h, 12 h, 1 d, 3 d, and 5 d postmortem. Shear force, pH, myofibril fragmentation index, AMPK activity, E3 ubiquitin ligase abundance, protein phosphorylation, and the ubiquitination levels of muscle samples were measured. The results demonstrated that the meat of samples in the tender group had a higher degradation of desmin and a lower phosphorylation level of desmin at 1 d compared with the tough group. The ubiquitination level of desmin, AMPK activity, and E3 ubiquitin ligase abundance in the tender group were noticeably higher than those in the tough group at 12 h. There was a negative correlation between the shear force and desmin degradation. The desmin degradation was negatively correlated with desmin phosphorylation and ubiquitination levels. The phosphorylation level of desmin was positively correlated with its ubiquitination. In summary, this study suggests that AMPK and E3 ubiquitin ligase concurrently play significant roles in regulating meat tenderness by regulating phosphorylation and ubiquitination in meat postmortem.

## 1. Introduction

Meat tenderness is considered to be an essential index for determining meat quality, which is a key concern of consumers [1]. Myofibrillar proteins constitute the predominant protein content in meat and its integrity is mainly responsible for the process of meat tenderization [2]. Desmin makes a notable contribution by linking the Z-disk to other cytoskeletal components, which establishes its association with the Z-disk and facilitates connections with adjacent cytoskeletal elements [3]. Li et al., 2017, observed that μ-calpain played an important role in desmin degradation postmortem, and that the structure of myofibrillar was destroyed which resulted in the enhancement of tenderness [4]. Thus, the degradation of myofibrillar proteins, especially desmin, is fundamental to the development of meat tenderization.

Phosphorylation and ubiquitination are pivotal protein posttranslational modifications (PTMs) that are involved in the formation of meat tenderness [5,6]. Protein phosphorylation has been confirmed to participate in regulating meat tenderness during postmortem storage through the phosphorylation of myofibrillar proteins [6,7]. Ubiquitination stands as another crucial posttranslational modification that can regulate protein degradation, and could also serve as a proxy for the degree of meat tenderization in postmortem muscles [8,9]. The ubiquitin proteasome in muscle cells may be activated to degrade myofibrillar proteins in the early postmortem period [10]. It has been hypothesized that the degradation of myofibrillar fibers is affected by the ubiquitinated proteins postmortem [11].

There is substantiated evidence suggesting that diverse posttranslational protein modifications may collaborate in an intricate or multifaceted way. The interaction between phosphorylation and acetylation could promote the dissociation of actomyosin and regulate glycolytic enzymes’ activity [6]. The ubiquitination and SUMOylation functions at adjacent sites were lost due to the absence of lysine residue acetylation sites [12]. Recent studies have exposed that protein phosphorylation and ubiquitination play a co-regulated part in protein degradation and muscle atrophy processes [13,14]. AMP-activated protein kinase (AMPK) and E3 ubiquitin ligase are recognized as major factors affecting protein phosphorylation and ubiquitination [15,16]. MAFbx and MuRF1 are two vital muscle-specific ubiquitin ligases, which were found to be involved in the ubiquitination of skeletal muscle [16,17]. Li et al., 2023, showed that the protein degradation could be promoted by the increased expressions of MuRF1 and MAFbx [18]. However, the co-regulation mechanism of phosphorylation and ubiquitination on protein degradation remains unknown. Therefore, the aim of this study was to investigate the possible effects of phosphorylation and ubiquitination of myofibrillar proteins on meat tenderness in mutton.

## 2. Materials and Methods

### 2.1. Sampling and Grouping

The *longissimus thoracis lumborum* (LTL) muscles from both sides of 64 sheep carcasses were collected rapidly after slaughtering. The carcasses were from sheep with the same age, gender, and feeding conditions. The samples were stored at 4 °C for 1 h, 12 h, 1 d, 3 d, and 5 d postmortem. The shear force of the 64 muscle samples was measured at 1 d postmortem and the results were sorted from largest to smallest. The 9 samples with the lowest shear force values were used as the high-tenderness group (tender), and the 9 samples with the highest shear force values were used as the low-tenderness group (tough) (*n* = 9).

### 2.2. Meat Quality Measurement

#### 2.2.1. pH

The pH meter (Testo205 pH meter, Lenzkirch, Germany) was calibrated using calibrate solutions with pH values of 4.0, 7.0, and 10.0. The pH values of each muscle sample were measured three times at 1 h, 12 h, 1 d, 3 d, and 5 d postmortem.

#### 2.2.2. Shear Force

The shear force was measured following the method of Holman et al., 2015, with few modifications [19]. The samples were heated to 71 °C in water bath using cooking bags until the core temperature reached 71 °C, and then cooled in cold water for 0.5 h. The muscle blocks were stored at 4 °C overnight and then cut into cuboidal strips of 4 × 1 × 1 cm. A tenderness meter (C-LM3, Northeast Agricultural University, Harbin, China) was used to measure the shear force three times.

### 2.3. Protein Phosphorylation and Ubiquitination Measurement

#### 2.3.1. Phosphorylation and Ubiquitination Levels of Myofibrillar Proteins

The myofibrillar proteins were obtained according to Ren et al., 2020, with some modifications [20]. Phosphorylation was detected by fluorescence staining. First, 15 μg of protein was loaded on a gel which consisted of 4% stacking gel and 10% separating gel, and then the test was run from 80 V to 120 V. Pro-Q and Ruby staining (Invitrogen, Eugene, OR, USA) were used for measuring phosphorylated protein and total protein, respectively [21]. The ratio of the band intensity of the phosphorylated protein to total protein was calculated as the relative protein phosphorylation level. Ubiquitination was examined by Western blotting. Proteins were separated by electrophoresis, and transferred to nitrocellulose membrane at 100 V for 120 min. We used 5% Nonfat Dry Milk (9999S, CST, Beverly, MA, USA) to block the membranes at room temperature for 60 min. The ubiquitination antibody (dilution 1:1000, 10201-2-AP, Proteintech, Wuhan, China) was used as the primary antibody and incubated at 4 °C overnight; then, HRP goat anti-rabbit IgG (dilution 1:5000, AS014, ABclonal, Wuhan, China) was used as the secondary antibody at room temperature for 90 min. ECL Substrate (1705062, Bio-Rad, Hercules, CA, USA) was used to visualize the membranes. The ratio of the band intensity of ubiquitinated protein to total protein was calculated as the relative protein ubiquitination level.

#### 2.3.2. Phosphorylation and Ubiquitination Levels of Desmin

Desmin was extracted following the method of IP/Co-IP kit (Thermo, Rockford, IL, USA). Phosphorylation and ubiquitination were examined by Western blotting. Proteins were separated by electrophoresis, and transferred to a nitrocellulose membrane at 350 mA for 90 min and 100 V for 120 min. We used 5% Nonfat Dry Milk (9999S, CST, Beverly, MA, USA) to block the membranes for 60 min. The phosphorylation antibody (dilution 1:1000, M210030S, Abmart, Shanghai, China) and ubiquitination antibody (dilution 1:1000, 10201-2-AP, Proteintech, Wuhan, China) were used as primary antibodies. HRP goat anti-rabbit IgG (dilution 1:5000, AS014, ABclonal, Wuhan, China) was used as the secondary antibody. ECL Substrate (1705062, Bio-Rad, Hercules, CA, USA) was used to visualize the membranes. The ratio of band intensity of phosphorylated desmin and total desmin was calculated as relative desmin phosphorylation level. The ratio of the band intensity of ubiquitinated desmin to total desmin was calculated as the relative desmin ubiquitination level.

### 2.4. Degradation of Desmin

The degradation of desmin was determined by SDS-PAGE and Western blot. Proteins were separated by electrophoresis, and transferred to nitrocellulose membrane with a voltage of 350 mA for 90 min. We used 5% Nonfat Dry Milk (9999S, CST, Beverly, MA, USA) to block the membranes for 60 min. The desmin rabbit antibody (dilution 1:1000, A0699, ABclonal, Wuhan, China) was used as the primary antibody. HRP goat anti-rabbit IgG (dilution 1:5000, AS014, ABclonal, Wuhan, China) was used as the secondary antibody. ECL Substrate (1705062, Bio-Rad, Hercules, CA, USA) was used to visualize the membranes. The ratio of the band intensity of degraded desmin to total desmin was calculated as the relative degradation of desmin.

### 2.5. Activity of AMPK

Proteins were extracted according to Veiseth et al., 2001, with some modifications [22]. First, 12 μg of protein was loaded on stain-free gel which consisted of 4% stacking gel and 10% separating gel and the test was run from 80 V to 120 V; then, the proteins were transferred to PVDF membrane at 60 V for 120 min. We used 5% Nonfat Dry Milk (9999S, CST, Beverly, MA, USA) to block the membranes for 60 min. The antiphospho-AMPKα (Thr172) (dilution 1:1000, 2535S, CST, Beverly, MA, USA) and AMPKα (dilution 1:1000, 2532S, CST, Beverly, MA, USA) were used as primary antibodies. HRP goat anti-rabbit IgG (dilution 1:5000, AS014, ABclonal, Wuhan, China) was used as the secondary antibody. ECL Substrate (1705062, Bio-Rad, Hercules, CA, USA) was used to visualize the membranes. The ratio of the band intensity of p-AMPK to total AMPK was calculated as the relative activity of AMPK.

### 2.6. Abundance of MuRF1 and MAFbx

About 0.1 g of the frozen samples was pulverized with 0.9 mL ice-cold PBS (pH 7.0) buffer. After centrifuging for 20 min at 4 °C, 3000× *g*, the supernatant was recovered. The abundance was detected by the MuRF1 ELISA KIT (MLBio, Shanghai, China) and MAFbx ELISA KIT (MLBio).

### 2.7. Statistical Analysis

The data were reported as means with standard errors and analyzed by ANOVA in SPSS Statistic 26.0 (IBM Corporation, New York, NY, USA). A repeated measure analysis of variance of the general linear model was used, in which the different tenderness groups and postmortem times were used as fixed factors. The significance among all means was determined using Duncan’s multiple range test (*p* < 0.05). Origin 2021 (OriginLab Co., Northampton, MA, USA) was used to test the correlation between shear force, the degradation of desmin, the phosphorylation and ubiquitination levels of the degradation, and enzyme activity according to Pearson’s method. The images were scanned using the ChemiDoc™ MP Imaging System (Bio-Rad, Hercules, CA, USA); the band intensities of electrophoresis and Western blot were evaluated and quantified by Quantity One 4.6.2 (Bio-Rad, Hercules, CA, USA).

## 3. Results

### 3.1. Meat Quality

The pH and shear force of mutton with different tenderness values are shown in Table 1. The shear force was significantly determined by tenderness group and postmortem time. According to the shear force of the mutton muscles at 1 d postmortem, there were significant differences between the two groups (*p* < 0.05), showing that the samples could be divided into a tender group and a tough group, and this grouping could be used for subsequent studies. Postmortem time had a significant effect on the pH, while group had an insignificant effect on it. In the tender group, the pH of the samples declined significantly from 1 h to 1 d (*p* < 0.05), but it remained consistent from 1 d until 5 d. In the tough group, the pH decreased significantly within 12 h (*p* < 0.05), and it remained stable until 5 d.

### 3.2. Phosphorylation and Ubiquitination Levels of Myofibrillar Proteins

**Figure 1:**
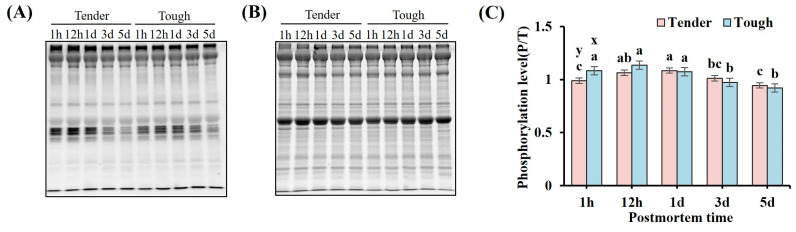
The phosphorylation level of the myofibrillar proteins in the tender and tough groups of mutton muscle stored at 4 °C for 5 d postmortem. (**A**) An image of Pro-Q diamond staining for phosphorylated myofibrillar proteins. (**B**) An image of SYPRO Ruby staining for total proteins. (**C**) The phosphorylation level of myofibrillar protein with different tenderness. (*n* = 9). Note: x and y represent significant differences between different groups at the same time points (*p* < 0.05). a–c represent significant differences between different times within the same group (*p* < 0.05).

The phosphorylation level of the myofibrillar proteins in the tender group at 1 h was significantly lower than that in the tough group (*p* < 0.05, Figure 1C). The phosphorylation level of the myofibrillar proteins in the tender group exhibited an initial increase from 1 h to 1 d, which was then followed by a decrease from 1 d to 5 d, reaching the highest value at 1 d (*p* < 0.05, Figure 1C). The myofibrillar proteins phosphorylation level in the tough group remained stable from 1 h to 1 d, but a significant decrease was observed at 3 d and the levels remained stable until 5 d (*p* < 0.05, Figure 1C).

**Figure 2:**
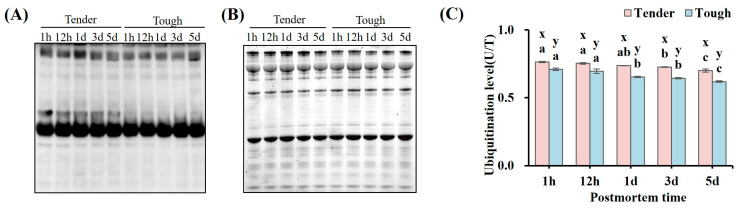
The ubiquitination level of the myofibrillar proteins in the tender and tough groups of mutton muscle stored at 4 °C for 5 d postmortem. (**A**) An image of the Western blot of ubiquitinated myofibrillar proteins in samples of different tenderness. (**B**) The ubiquitination level of the myofibrillar proteins with different tenderness. (**C**) The ubiquitination level of the myofibrillar protein with different tenderness. (*n* = 9). Note: x and y represent significant differences between different groups at the same time points (*p* < 0.05). a–c represent significant differences between different times within the same group (*p* < 0.05).

The ubiquitination level of the myofibrillar proteins in the tender and tough groups of mutton muscle stored at 4 °C for 5 d postmortem are shown in Figure 2. The ubiquitination level of the myofibrillar proteins in the tender group was significantly higher than that in the tough group at all time points (*p* < 0.05). The ubiquitination level of the myofibrillar proteins in the tough group decreased significantly at 1 d (*p* < 0.05), while it was identified that, in the tender group, it decreased significantly at 3 d (*p* < 0.05), which was later than in the tough group.

### 3.3. The Degradation, Phosphorylation, and Ubiquitination of Desmin

**Figure 3:**
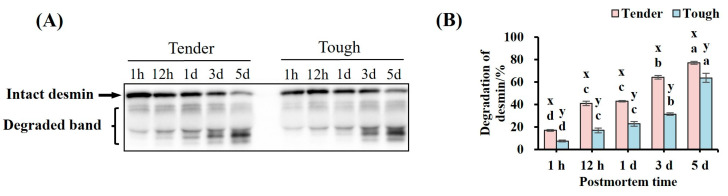
The degradation of desmin in tender and tough groups of mutton muscle stored at 4 °C for 5 d postmortem. (**A**) An image of a Western blot showing the abundance of desmin. (**B**) The degradation of desmin in samples of different tenderness. (*n* = 9). Note: x, y represent significant differences between different groups at the same time points (*p* < 0.05). a–d represent significant differences between different times within the same group (*p* < 0.05).

It can be observed that there was a significant difference between the myofibrillar protein degradation in the two groups (Figure 1B). Further, the expression of the main bands of desmin decreased gradually with the extension of postmortem time, while the expression of degraded fragments of desmin increased gradually (Figure 3A). The desmin degradation in the tender group was higher than that in the tough group at all time points (*p* < 0.05, Figure 3B). It was found that there was a significant expression of degraded fragments of desmin at 1 h in both groups (Figure 3A). The degradation of desmin in both groups reached the maximum value at 5 d (*p* < 0.05, Figure 3B).

**Figure 4:**
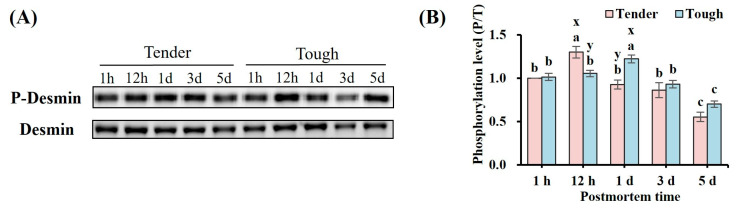
The phosphorylation level of desmin in tender and tough groups of mutton muscle stored at 4 °C for 5 d postmortem. (**A**) An image of the Western blot of the phosphorylation level of desmin. (**B**) The phosphorylation level of desmin in samples with different tenderness. (*n* = 9). Note: x and y represent significant differences between different groups at the same time points (*p* < 0.05). a–c represent significant differences between different times within the same group (*p* < 0.05).

The phosphorylation levels of desmin in the tender and tough groups of mutton muscle stored at 4 °C for 5 d postmortem are shown in Figure 4. There was a trend showing that the phosphorylation level of desmin in both groups increased firstly and then decreased postmortem. In contrast to the tough group, the desmin phosphorylation level in the tender group was higher at 12 h, while in the tough group it was significantly higher than that of the tender group at 1 d (*p* < 0.05). The desmin phosphorylation level in the tender group was significantly higher at 12 h than at the other timepoints, while in the tough group it reached its highest value at 1 d (*p* < 0.05). It can be observed that there was a decline at 5 d in both groups (*p* < 0.05).

**Figure 5:**
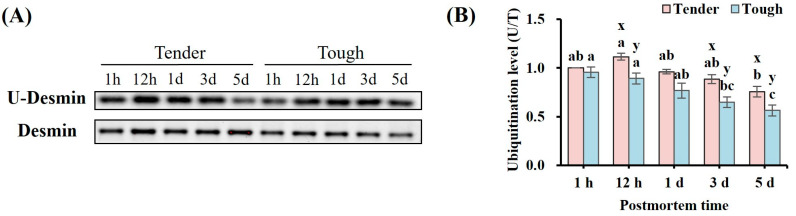
The ubiquitination level of desmin in tender and tough groups of mutton muscle stored at 4 °C for 5 d postmortem. (**A**) The image of Western blot of ubiquitination level of desmin. (**B**) The ubiquitination level of desmin in samples with different tenderness. (*n* = 9). Note: x and y represent significant differences between different groups at the same time points (*p* < 0.05). a–c represent significant differences between different times within the same group (*p* < 0.05).

The ubiquitination levels of desmin in the tender and tough groups of mutton muscle stored at 4 °C for 5 d postmortem are shown in Figure 5. The desmin ubiquitination level in the tender group was significantly higher than that in the tough group at 12 h, 3 d, and 5 d (*p* < 0.05). The ubiquitination level of desmin in the tender group increased firstly and then decreased with postmortem time. The desmin ubiquitination level in the tender group reached highest value at 12 h (*p* < 0.05). While it decreased gradually and declined significantly at 3 d compared with 12 h in the tough group (*p* < 0.05).

### 3.4. AMPK Activity and MuRF1 and MAFbx Abundance

**Figure 6:**
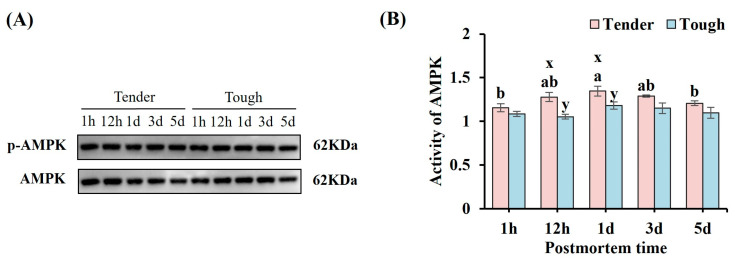
The activity of AMPK in the tender and tough groups of mutton muscle stored at 4 °C for 5 d postmortem. (**A**) An image of the Western blot of AMPK with different tenderness. (**B**) The activity of AMPK in samples of different tenderness. (*n* = 9). Note: x and y represent significant differences between different groups at the same time points (*p* < 0.05). a and b represent significant differences between different times within the same group (*p* < 0.05).

The activity of AMPK in the tender and tough groups of mutton muscle stored at 4 °C for 5 d postmortem is shown in Figure 6. The AMPK activity in the tender group was higher at 12 h and 1 d than that in the tough group (*p* < 0.05). The activity of AMPK in the tender group increased first, then reached maximum value at 1 d, and decreased significantly at 5 d (*p* < 0.05), while it showed a stable trend in the tough group over 5 d (*p* > 0.05).

**Figure 7:**
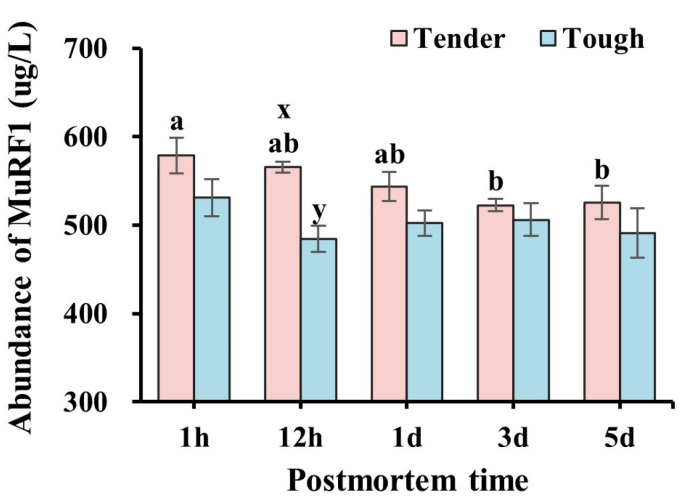
The abundance of MuRF1 in the tender and tough groups of mutton muscle stored at 4 °C for 5 d postmortem. (*n* = 9). Note: x and y represent significant differences between different groups at the same time points (*p* < 0.05). a and b represent significant differences between different times within the same group (*p* < 0.05).

The abundance of MuRF1 in the tender and tough groups of mutton muscle stored at 4 °C for 5 d postmortem is shown in Figure 7. The abundance of MuRF1 in the tender group was higher than that in the tough group at 12 h (*p* < 0.05). In the tender group, the abundance of MuRF1 decreased significantly at 3 d compared with 1 h (*p* < 0.05), while it can be observed that there were insignificant differences at all time points in the tough group (*p* > 0.05).

**Figure 8:**
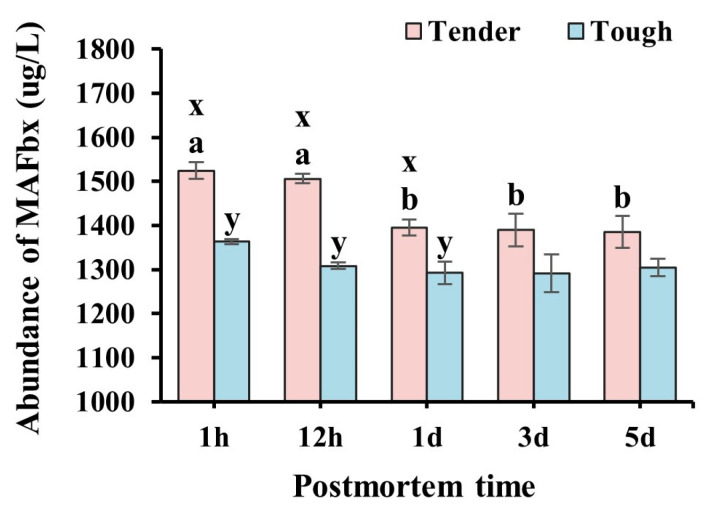
The abundance of MAFbx in the tender and tough groups of mutton muscle stored at 4 °C for 5 d postmortem. (*n* = 9). Note: x and y represent significant differences between different groups at the same time points (*p* < 0.05). a and b represent significant differences between different times within the same group (*p* < 0.05).

The abundance of MAFbx in the tender and tough groups of mutton muscle stored at 4 °C for 5 d postmortem is shown in Figure 8. The abundance of MAFbx in the tender group was higher than that of the tough group at 1 h, 12 h, and 1 d (*p* < 0.05). The abundance of MAFbx in the tender group showed a decreasing trend with postmortem time, which decreased significantly at 1 d and continued to be stable until 5 d (*p* < 0.05), while it showed a stable trend in the tough group over 5 d (*p* > 0.05).

### 3.5. Correlation Analysis of the Degradation and PTMs of Desmin and Enzyme Activity

**Figure 9:**
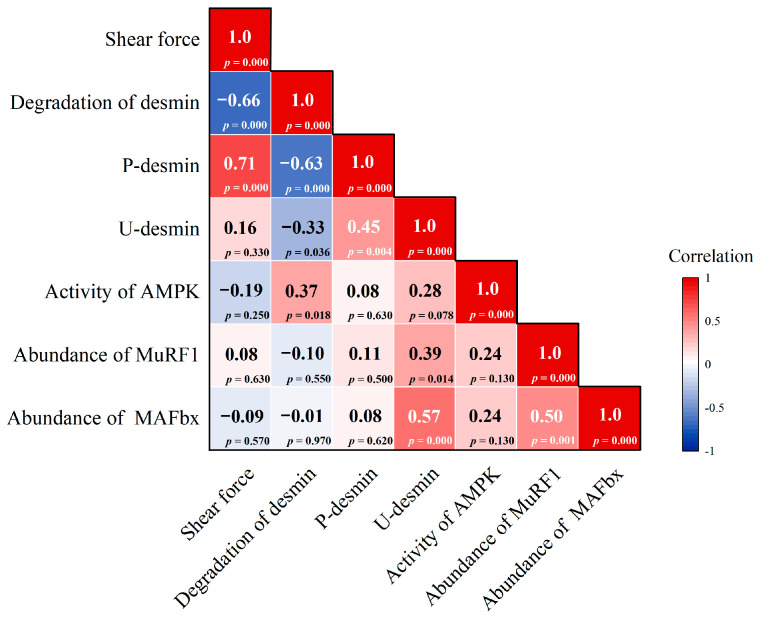
Correlation analysis of shear force, degradation of desmin, phosphorylation and ubiquitination levels of degradation and enzyme activity of mutton muscle. Note: Red color represents positive correlation, blue color represents negative correlation. Each rectangle indicates *r*-value (Pearson correlation coefficient for a pair of variables) and *p*-value.

The results of the correlation analysis between shear force, degradation of desmin, the phosphorylation and ubiquitination levels of degradation, and enzyme activity of mutton muscle are shown in Figure 9. The result of the correlation analysis showed that the shear force was negatively correlated with the degradation of desmin (*p* < 0.05). The degradation of desmin was negatively correlated with the phosphorylation and ubiquitination levels of desmin, while the AMPK activity was positively correlated with desmin degradation (*p* < 0.05). The desmin phosphorylation level showed a positive correlation with its ubiquitination level (*p* < 0.05). In addition, the abundance of MuRF1 and MAFbx had a positive correlation with the ubiquitination level of desmin (*p* < 0.05).

## 4. Discussion

Shear force is the main index that reflects meat tenderness, and meat tenderness is closely related to the degradation of myofibrillar proteins [23]. In this study, the shear force in the tender group reached its maximum value at 12 h and then decreased, while it reached its maximum value at 1 d in the tough group, which suggested that in the tender group the myofibrillar skeleton was damaged earlier than in the tough group and the tenderness was improved with the extension of postmortem time. Desmin is well known as a symbol of proteolysis, which has the function of attaching to the Z-disk [3,24]. Desmin degradation was a potential key factor affecting meat tenderness [24]. In this study, the desmin degradation result showed that it was initiated at 1 h postmortem in both groups. In the tender group, it was observably higher at all time points than that in the tough group. This result was similar to a previous result which suggested that the degradation of desmin symbolized the destruction of muscle structure, thus promoting meat tenderness in the early postmortem period [25]. Furthermore, the significant correlation between the shear force and desmin degradation indicated that the degradation of desmin could represent the breakdown of the transverse connections between sarcomere and act as a symbol of tenderness.

Compared with the tough group, it was clear that in the tender group the myofibrillar proteins ubiquitination level was higher at all time points, which indicated that ubiquitination might significantly contribute to meat tenderization. The ubiquitination level of desmin showed a negative correlation with its degradation, which indicated that ubiquitination could negatively impact meat tenderness. Gao et al., 2022, observed that the E3 ubiquitin ligase is essential to maintain the degradation of proteins [26]. MuRF1 and MAFbx are proprietary in muscle and may be the key factors that regulate ubiquitination in postmortem meat, but their functions have not been reported in depth [16,17]. A previous study suggested that MuRF1 positively regulated skeletal muscle atrophy and protein degradation [18]. Gomes et al., 2001, reported that desmin could be ubiquitinated by MAFbx [27]. The consequent results showed that compared with the tough group, the abundances of MuRF1 and MAFbx in the tender group were notably higher in the early postmortem period. There was a strong positive correlation between the abundance of MuRF1 and MAFbx and the desmin ubiquitination level, which suggested that MuRF1 and MAFbx might be key factors in regulating the protein ubiquitination level postmortem. These results demonstrated that both of them were important contributors to meat tenderness through the regulation of protein ubiquitination. Furthermore, the ubiquitin proteasome system constitutes a significant component of the protein degradation pathway, which is instrumental in controlling the degradation of skeletal muscle proteins [28]. In another study, the myofibrillar protein degradation levels were effectively inhibited by adding MG-132 (a proteasome inhibitor), which indicated that proteasome might be essential for the degradation of proteins in postmortem meat [11]. Therefore, it can be speculated that the ubiquitinated desmin was degraded by the ubiquitin proteasome system.

Protein phosphorylation has been identified as one of the key factors influencing myofibrillar proteins degradation, consequently affecting meat tenderness [5,21]. Based on the correlation analysis, the results indicated a correlation, showing that the desmin degradation was affected by desmin phosphorylation and AMPK activity. Compared with the tough group, the desmin phosphorylation level in the tender group was observably lower at 1 d when the shear force in the tender group was also significantly lower. A previous study also found that, with a lower phosphorylation level, desmin degradation was inhibited [29]. However, in the present study, an insignificant difference was observed in the myofibrillar protein phosphorylation level between the two groups, except at 1 h. It can be speculated that proteins with different modification levels might co-regulate protein degradation during the postmortem period [30]. AMPK is a crucial kinase that regulates energy homeostasis and plays a key role in diverse signal transduction pathways within the body [31,32]. It has been shown that AMPK could regulate meat quality by participating in the regulation of multiple energy metabolic pathways [15,33]. Furthermore, it has been demonstrated that AMPK could participate in protein degradation by regulating protein phosphorylation. Although in this study AMPK activity was not strongly correlated with the phosphorylation level of desmin, it was positively correlated with desmin degradation, and we speculated that this might be due to the fact that other protein kinases played a major role in regulating the phosphorylation level, AMPK might be involved in other pathways that regulate protein degradation.

Recent studies have shown that there is a crosstalk between phosphorylation and ubiquitination in medicine and molecular biology [13]. For example, F-Box Protein 22 can recognize the phosphorylation of Bcl-2-associated athanogene 3 (BAG3) at the S377 site, catalyzed by extracellular regulated protein kinases, and then promote the ubiquitination and the degradation of BAG3 [34]. Han et al., 2019, demonstrated that AMPK phosphorylation could inhibit the interaction with glycoprotein 78, and then restrain its ubiquitination and degradation [33]. The phosphorylation of the C-terminal T912 site of RBOHD could promote protein ubiquitination and degradation and decrease protein abundance [35]. In this study, we also found that there was a crosstalk between phosphorylation and ubiquitination; the correlation analysis indicated that desmin’s phosphorylation was positively correlated with its ubiquitination. Shenhav et al., 2020, found that the phosphorylation of desmin could facilitate its ubiquitination and then the desmin was degraded [36]. Some studies have suggested that AMPK might participate in regulating the expression levels of MuRF1 and MAFbx [37]. Therefore, the relationship between AMPK and E3 ubiquitin ligase might contribute to the interaction of protein phosphorylation and ubiquitination during protein degradation postmortem. In summary, the co-effects of phosphorylation and ubiquitination on desmin degradation could provide a better insight into the transformation from muscle to meat, and they potentially exhibited a synergistic effect in the early postmortem period.

## 5. Conclusions

This study showed that the degradation of desmin was possibly affected by its phosphorylation and ubiquitination postmortem. There was a positive correlation between the phosphorylation level of desmin and its ubiquitination level. AMPK, MuRF1, and MAFbx might be the key factors influencing this interaction, and they consequently co-regulate protein degradation consequently. In general, this research provided a novel possible way to understand the mechanism of protein degradation during postmortem from the perspective of the cooperation of phosphorylation and ubiquitination.

## Figures and Tables

**Table 1:** The pH and shear force in tender and tough groups of mutton muscle stored at 4 °C for 5 d postmortem.

	Groups	Postmortem Time					*p*-Values		
		1 h	12 h	1 d	3 d	5 d	Times	Groups	Interaction
pH	Tender	7.19 ± 0.05 ^aX^	6.23 ± 0.073 ^bX^	5.75 ± 0.01 ^c^	5.72 ± 0.02 ^cY^	5.74 ± 0.03 ^cY^	<0.001	0.980	0.003
	Tough	6.86 ± 0.10 ^aY^	6.08 ± 0.04 ^bY^	5.92 ± 0.09 ^b^	5.87 ± 0.05 ^bX^	5.90 ± 0.05 ^bX^			
Shear force	Tender	65.21 ± 5.38 ^a^	70.48 ± 5.41 ^a^	63.19 ± 2.23 ^aY^	43.90 ± 7.27 ^b^	21.60 ± 0.82 ^cY^	0.002	0.003	0.015
	Tough	61.44 ± 3.01 ^b^	63.40 ± 2.87 ^b^	86.15 ± 1.76 ^aX^	63.56 ± 5.12 ^b^	53.57 ± 6.50 ^bX^			

Note: Within the same row, different letters, a–c, represent significant differences between times within the same group (*p* < 0.05). Within the same column, different letters, X, Y, represent significant differences between groups within the same time (*p* < 0.05).

## Data Availability

The original contributions presented in the study are included in the article/Supplementary Materials, further inquiries can be directed to the corresponding author.

## Supplementary Materials

The following supporting information can be downloaded at: https://www.mdpi.com/article/10.3390/foods14020184/s1, Figure S1. The raw image of degradation of desmin in tender and tough groups of mutton muscle stored at 4 °C for 5 d postmortem. Figure S2. The raw images of phosphorylation level of desmin in tender and tough groups of mutton muscle stored at 4 °C for 5 d postmortem. Figure S3. The raw images of ubiquitination level of desmin in tender and tough groups of mutton muscle stored at 4 °C for 5 d postmortem. Figure S4. The raw images of activity of AMPK in tender and tough groups of mutton muscle stored at 4 °C for 5 d postmortem.

## References

[1] Warner R.D., Wheeler T.L., Ha M., Li X., Bekhit A.E.D., Morton J., Vaskoska R., Dunshea F.R., Liu R., Purslow P. (2022). Meat tenderness: Advances in biology, biochemistry, molecular mechanisms and new technologies. Meat Sci..

[2] Ma D., Brad-Kim Y.H. (2020). Proteolytic changes of myofibrillar and small heat shock proteins in different bovine muscles during aging: Their relevance to tenderness and water-holding capacity. Meat Sci..

[3] Huff-Lonergan E., Zhang W.G., Lonergan S.M. (2010). Biochemistry of postmortem muscle—Lessons on mechanisms of meat tenderization. Meat Sci..

[4] Li Z., Li X., Gao X., Shen Q.W., Du M.T., Zhang D.Q. (2017). Phosphorylation prevents in vitro myofibrillar proteins degradation by μ-calpain. Food Chem..

[5] Li X., Zhang D.Q., Ren C., Bai Y.Q., Ljaz M., Hou C.L., Chen L. (2021). Effects of protein posttranslational modifications on meat quality: A review. Compr. Rev. Food Sci. Food Saf..

[6] Ren C., Li X., Bai Y.Q., Schroyen M., Zhang D.Q. (2022). Phosphorylation and acetylation of glycolytic enzymes cooperatively regulate their activity and lamb meat quality. Food Chem..

[7] Li Z., Li M., Du M.T., Shen Q.W., Zhang D.Q. (2018). Dephosphorylation enhances postmortem degradation of myofibrillar proteins. Food Chem..

[8] Cockram P.E., Kist M., Prakash S., Chen S.H., Wertz I.E., Vucic D. (2021). Ubiquitination in the regulation of inflammatory cell death and cancer. Cell Death Differ..

[9] Picard B., Gagaoua M., Colgrave M.L. (2017). Proteomic investigations of beef tenderness. Proteomics in Food Science.

[10] Nyam-Osor P., Jayawardana B., Aro J., Shimada K., Fukushima M., Sekikawa M. (2009). Identification of ubiquitin conjugated protein phosphatase inhibitor 1 from postmortem bovine muscle. Food Chem..

[11] Liu Y., Du M.T., Li X., Chen L., Shen Q.W., Tian J.W., Zhang D.Q. (2016). Role of the ubiquitin-proteasome pathway on proteolytic activity in postmortem proteolysis and tenderisation of sheep skeletal muscle. Int. J. Food Sci. Technol..

[12] Gupta R., Kumar P. (2021). Computational analysis indicates that PARP1 acts as a histone deacetylases interactor sharing common lysine residues for acetylation, ubiquitination, and SUMOylation in Alzheimer’s and Parkinson’s disease. ACS Omega.

[13] Filipčík P., Curry J.R., Mace P.D. (2017). When worlds Collide-Mechanisms at the interface between phosphorylation and ubiquitination. J. Mol. Biol..

[14] Goldbraikh D., Neufeld D., Eid-Mutlak Y., Lasry I., Gilda J.E., Parnis A., Cohen S. (2020). USP1 deubiquitinates Akt to inhibit PI3K-Akt-FoxO signaling in muscle during prolonged starvation. EMBO Rep..

[15] Shen Q.W., Means W.J., Thompson S.A., Underwood K.R., Zhu M.J., McCormick R.J., Ford S.P., Du M. (2006). Pre-slaughter transport, AMP-activated protein kinase, glycolysis, and quality of pork loin. Meat Sci..

[16] Clarke B.A., Drujan D., Willis M.S., Murphy L.O., Corpina R.A., Burova E., Rakhilin S.V., Stitt T.N., Patterson C., Latres E. (2007). The E3 ligase MuRF1 degrades myosin heavy chain protein in dexamethasone-treated skeletal muscle. Cell Metab..

[17] Bodine S.C., Latres E., Baumhueter S., Lai V.K., Nunez L., Clarke B.A., Poueymirou W.T., Panaro F.J., Na E., Dharmarajan K. (2001). Identification of ubiquitin ligases required for skeletal muscle atrophy. Science.

[18] Li J.P., Hu Y.Q., Li J.J., Wang H.T., Wu H.Y., Zhao C.C., Tan T., Zhang L., Zhu D., Liu X. (2023). Loss of MuRF1 in Duroc pigs promotes skeletal muscle hypertrophy. Transgenic Res..

[19] Holman B.W.B., Alvarenga T.I.R.C., Ven R.J., Hopkins D.L. (2015). A comparison of technical replicate (cuts) effect on lamb Warner-Bratzler shear force measurement precision. Meat Sci..

[20] Ren C., Hou C.L., Li Z., Li X., Bai Y.Q., Zhang D.Q. (2020). Effects of temperature on protein phosphorylation in postmortem muscle. J. Sci. Food Agric..

[21] Chen L.J., Li X., Ni N., Liu Y., Chen L., Wang Z.Y., Shen Q.W., Zhang D.Q. (2016). Phosphorylation of myofibrillar proteins in post-mortem ovine muscle with different tenderness. J. Sci. Food Agric..

[22] Veiseth E., Shackelford S.D., Wheeler T.L., Koohmaraie M. (2001). Effect of postmortem storage on μ-calpain and m-calpain in ovine skeletal muscle. J. Anim. Sci..

[23] Hopkins D.L., Littlefield P.J., Thompson J.M. (2000). A research note on factors affecting the determination of myofibrillar fragmentation. Meat Sci..

[24] Malva A., Gagaoua M., Santillo A., Palo P.D., Sevi A., Albenzio M. (2022). First insights about the underlying mechanisms of Martina Franca donkey meat tenderization during aging: A proteomic approach. Meat Sci..

[25] Muroya S., Ertbjerg P., Pomponio L., Christensen M. (2010). Desmin and troponin T are degraded faster in type IIb muscle fibers than in type I fibers during postmortem aging of porcine muscle. Meat Sci..

[26] Gao S., Zhang G.H., Zhang Z.C., Zhu J.Z., Li L., Zhou Y., Rodney G.G., Abo-Zahrah R.S., Anderson L., Garcia J.M. (2022). UBR2 targets myosin heavy chain IIb and IIx for degradation: Molecular mechanism essential for cancer-induced muscle wasting. Proc. Natl. Acad. Sci. USA.

[27] Gomes M.D., Lecker S.H., Jagoe R.T., Navon A., Goldberg A.L. (2001). Atrogin-1, a muscle-specific F-box protein highly expressed during muscle atrophy. Proc. Natl. Acad. Sci. USA.

[28] Bilodeau P.A., Coyne E.S., Wing S.S. (2016). The ubiquitin proteasome system in atrophying skeletal muscle: Roles and regulation. Am. J. Physiol. Cell Physiol..

[29] Ren C., Song X.B., Dong Y., Hou C.L., Chen L., Wang Z.Y., Schroyen M., Zhang D.Q. (2023). Protein phosphorylation induced by pyruvate kinase M2 inhibited myofibrillar protein degradation in post-mortem muscle. J. Agric. Food Chem..

[30] Ren C., Chen L., Bai Y.Q., Hou C.L., Li X., Schroyen M., Zhang D.Q. (2024). Comparative effects of phosphorylation and acetylation on glycolysis and myofibrillar proteins degradation in postmortem muscle. Int. J. Biol. Macromol..

[31] Hardie D.G., Ross F.A., Hawley S.A. (2012). AMPK: A nutrient and energy sensor that maintains energy homeostasis. Nat. Rev. Mol. Cell Biol..

[32] Keerthana C.K., Rayginia T.P., Shifana S.C., Anto N.P., Kalimuthu K., Isakov N., Anto R.J. (2023). The role of AMPK in cancer metabolism and its impact on the immunomodulation of the tumor microenvironment. Front. Immunol..

[33] Han Y.M., Hu Z.M., Cui A.Y., Liu Z.S., Ma F.J., Xue Y.Q., Liu Y.X., Zhang F.F., Zhao Z.H., Yu Y.Y. (2019). Dost-translational regulation of lipogenesis via AMPK-dependent phosphorylation of insulin-induced gene. Nat. Commun..

[34] Liu P., Cong X.J., Liao S.J., Jia X.L., Wang X.M., Dai W., Zhai L.H., Zhao L., Ji J., Ni D. (2022). Global identification of phospho-dependent SCF substrates reveals a FBXO22 phosphodegron and an ERK-FBXO22-BAG3 axis in tumorigenesis. Cell Death Differ..

[35] Lee D., Lal N.K., Lin Z.J.D., Ma S.S., Liu J., Castor B., Toruño T., Dinesh-Kumar S.P., Coaker G. (2020). Regulation of reactive oxygen species during plant immunity through phosphorylation and ubiquitination of RBOHD. Nat. Commun..

[36] Shenhav C. (2020). Role of calpains in promoting desmin filaments depolymerization and muscle atrophy. Biochim. Biophys. Acta Mol. Cell Res..

[37] Vilchinskaya N.A., Rozhkov S.V., Turtikova O.V., Mirzoev T.M., Shenkman B.S. (2023). AMPK phosphorylation impacts apoptosis in differentiating myoblasts isolated from atrophied rat soleus muscle. Cells.

