# Supplementary material for: Revealing the Mechanism of Protein Degradation in Postmortem Meat: The Role of Phosphorylation and Ubiquitination
Source: Foods. 2025 Jan 9;14(2):184. doi: 10.3390/foods14020184 (PMC11764534; doi:10.3390/foods14020184)
Supplement: Supplementary file 1 [file foods-14-00184-s001.zip › foods-3381470-supplementary.pdf]

Supplementary information

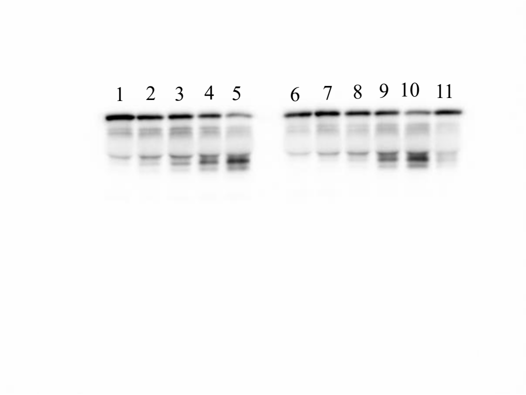

Figure S1. The raw image of degradation of desmin in tender and tough groups of mutton muscle stored at 4 °C for 5 d postmortem. 1-5 represent samples of 1 h, 12 h, 1 d, 3 d and 5 d in the tender group, 6-10 represent samples of 1 h, 12 h, 1 d, 3 d and 5 d in the tough group, 11 represents standard sample.

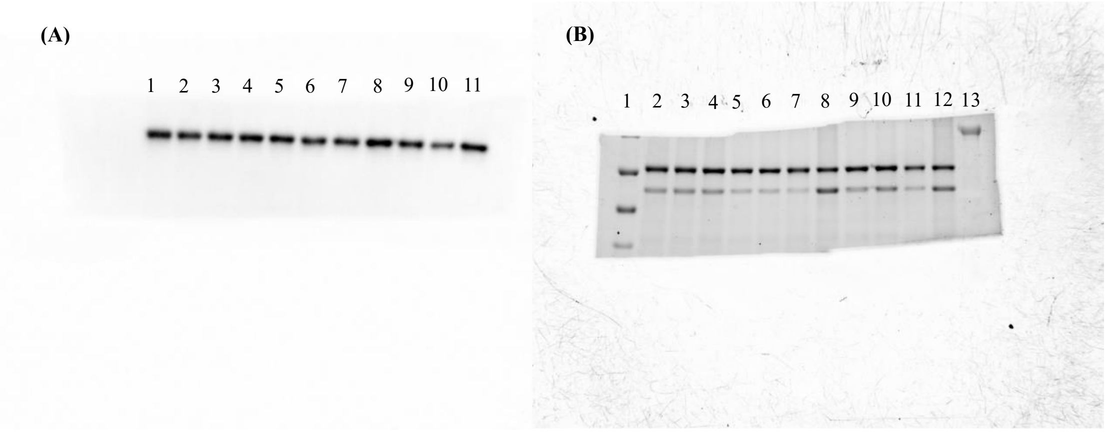

Figure S2. The raw images of phosphorylation level of desmin in tender and tough groups of mutton muscle stored at 4 °C for 5 d postmortem. (A) The image of western blotting of phosphorylation level of desmin. 1 represents standard sample, 2-6 represent samples of 1 h, 12 h, 1 d, 3 d and 5 d in the tender group, 7-11 represent samples of 1 h, 12 h, 1 d, 3 d and 5 d in the tough group. (B) The image of western blotting of total desmin. 1 and 13 represent marker, 2 represents standard sample, 3-7 represent samples of 1 h, 12 h, 1 d, 3 d and 5 d in the tender group, 8-12 represent samples of 1 h, 12 h, 1 d, 3 d and 5 d in the tough group.

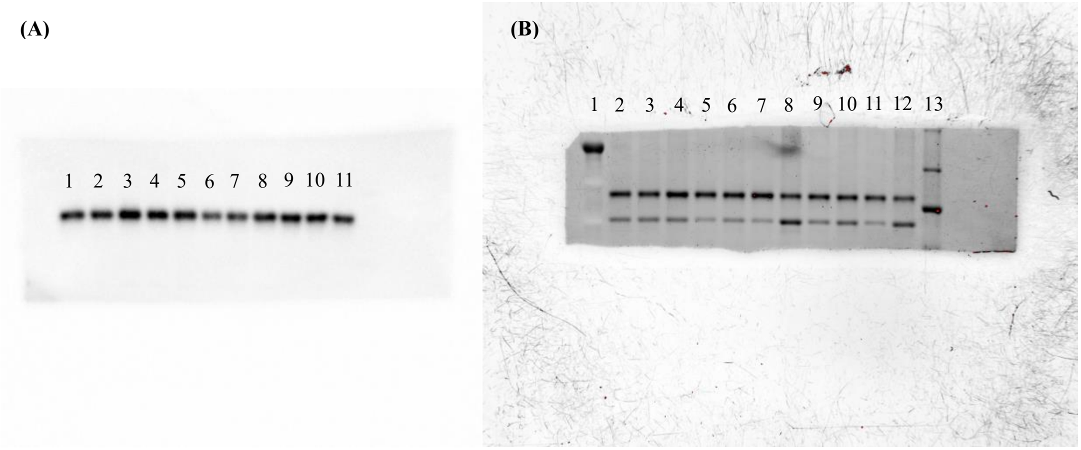

Figure S3. The raw images of ubiquitination level of desmin in tender and tough groups of mutton muscle stored at 4 °C for 5 d postmortem. (A) The image of western blotting of ubiquitination level of desmin. 1 represents standard sample, 2-6 represent samples of 1 h, 12 h, 1 d, 3 d and 5 d in the tender group, 7-11 represent samples of 1 h, 12 h, 1 d, 3 d and 5 d in the tough group. (B) The image of western blotting of total desmin. 1 and 13 represent marker. 2 represents standard sample, 3-7 represent samples of 1 h, 12 h, 1 d, 3 d and 5 d in the tender group, 8-12 represent samples of 1 h, 12 h, 1 d, 3 d and 5 d in the tough group.

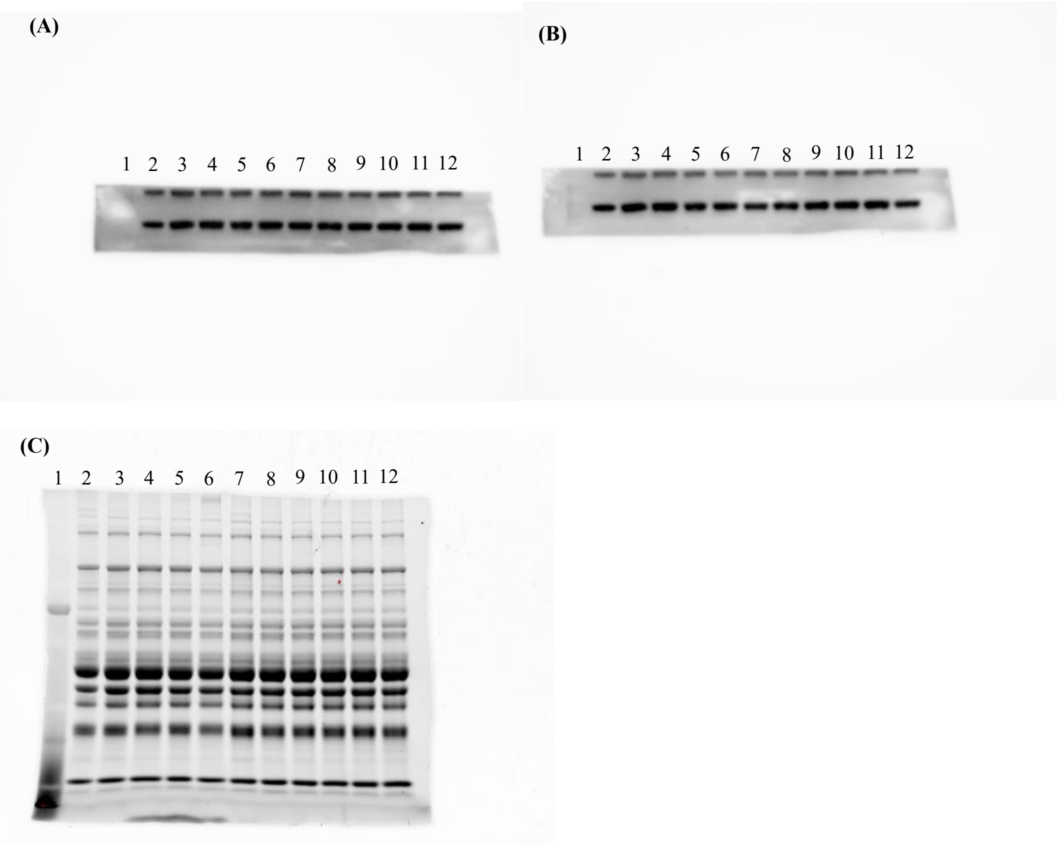

Figure S4. The raw images of activity of AMPK in tender and tough groups of mutton muscle stored at 4 °C for 5 d postmortem. (A) The image of western blotting of p-AMPK. 1 represents marker. 2 represents standard sample, 3-7 represent samples of 1 h, 12 h, 1 d, 3 d and 5 d in the tender group, 8-12 represent samples of 1 h, 12 h, 1 d, 3 d and 5 d in the tough group. (B) The image of western blotting of AMPK. 1 represents marker. 2 represents standard sample, 3-7 represent samples of 1 h, 12 h, 1 d, 3 d and 5 d in the tender group, 8-12 represent samples of 1 h, 12 h, 1 d, 3 d and 5 d in the tough group. (C) The image of SDS-PAGE of total protein. 1 represents marker. 2 represents standard sample, 3-7 represent samples of 1 h, 12 h, 1 d, 3 d and 5 d in the tender group, 8-12 represent samples of 1 h, 12 h, 1 d, 3 d and 5 d in the tough group.

Note: Marker and standard sample lines are not shown in the figures of the main article.
